# Benefits of a Natural Dietary Supplement for Tinnitus: An Observational Prospective Exploratory Study

**DOI:** 10.3390/audiolres16020048

**Published:** 2026-03-24

**Authors:** Massiel Cepeda Uceta, Estela Lladó-Carbó, Raidili Mateo Montero, Catalina Villa Jurado, Montserrat Virumbrales, Iván Domènech Juan

**Affiliations:** 1 AMiQ-IMO Otorhinolaryngology and Maxillofacial Surgery, C/Josep Maria Lladó 3, 08035 Barcelona, Spain; 2 Neurotoc, C/Padilla 327-329, 08025 Barcelona, Spain; 3 Department of Medicine, School of Medicine and Health Sciences, Universitat Internacional de Catalunya (UIC), 08017 Barcelona, Spain

**Keywords:** tinnitus, quality of life, anxiety, dietary supplement, L-theanine, melatonin, GABA, *Ginko biloba*, minerals, B vitamins

## Abstract

**Background/Objectives**: The objective of the study was to assess the benefits on quality of life (QoL) of a natural-based dietary supplement in patients with tinnitus. **Methods**: An observational, prospective and exploratory study was conducted in 30 patients (mean age 50.7 years) diagnosed with tinnitus. The dietary supplement (Otocalm^®^) contained L-theanine, *Gingko biloba*, melatonin, GABA, zinc, selenium and vitamins B3, B6 and B12, and was administered for 90 consecutive days. Clinical assessment included tone verbal audiometry, the Tinnitus Handicap Inventory (THI), the Goldberg anxiety and depression scale (GADS), and a 0–10 mm visual analogue scale (VAS) to score the intensity of tinnitus. **Results**: The mean THI score decreased from 40.8 at baseline to 30.9 at the end of the study (*p* = 0.012), and the percentage of patients with THI grade 1 (no handicap) increased from 3.3% to 20%. The mean anxiety score decreased from 4.7 to 3.0 (*p* = 0.006), and the percentage of patients scoring ≥ 4 in the GADS decreased from 63.3% to 33.3%. Changes in VAS scores and verbal tone audiometry were not observed. A decrease in the mean frequency of tinnitus from 2417.4 Hz to 1603.3 Hz (*p* = 0.519) was found. The product was safe and well-tolerated. **Conclusions**: The administration of a natural-based dietary supplement composed of L-theanine, *Ginkgo biloba*, melatonin, GABA, zinc, selenium, and group B vitamins during 90 days in patients with tinnitus was associated with a significant increase in QoL by reducing tinnitus-associated handicap and anxiety.

## 1. Introduction

Tinnitus, the perception of sound in the absence of an outside source, is a complex multifactorial problem that affects more than 740 million adults globally [1] and can be considered a disability when it substantially and persistently impairs daily activities and quality of life [2]. Tinnitus defined as the psychosensory phenomenon experienced in the auditory cortex can be subjective (the most common form in 95% of cases) and objective (with a prevalence between 5 and 10%) and is associated with different causes and a wide range of comorbidities, being loud noise, hearing loss, aging, temporomandibular joint dysfunction, ototoxic drugs, and stress/anxiety some of the most well recognized etiologies [3,4]. However, tinnitus is a heterogeneous condition with patients presenting a variety of overlapping symptoms and phenotypes related to the auditory component and the associated suffering characterized by psychological distress, sleep problems, concentration difficulties, behavioral changes, and functional impairment [5,6]. Genetic contribution to better identification of phenotypes has been a focus of increasing interest in recent tinnitus research [7,8].

In clinical practice, diagnosis includes a complete medical history and otorhinolaryngological evaluation to assess the etiology of tinnitus, often in association with other tests, such as pure tone audiometry, impedance audiometry, otoacoustic emissions, radiological imaging, evaluation of the activity of cortical central regions, and further analysis of tinnitus severity in terms of distress and impact depending on the presumed diagnosis and associated symptoms. It has been shown that quantitative electroencephalographic (EEG) activity differs in subjects with and without tinnitus [9] and that connectivity features of EEG (e.g., power spectral density and rhythm signals) can be used as biomarkers for an efficient and fast diagnostic method for chronic tinnitus [10]. The P300 component of the late auditory evoked potentials, an objective marker of cognitive potential, also showed significant increases in P300 latency and decreases in P300 amplitude in tinnitus patients as compared with controls, suggesting a link between tinnitus and cognition [11,12]. Due to the variety of symptoms, a multidisciplinary diagnostic approach using at least one measure of tinnitus-related disability is recommended [13].

There is no cure for tinnitus, and treatment modalities are aimed at controlling underlying disorders and suppressing or lessening the awareness of tinnitus and its impact on quality of life. Tinnitus treatments, especially in chronic cases, can be disappointing due to the myriad of etiologies and complex pathogenetic mechanisms. In a systematic review of the most important 10 guidelines published between 2011 and 2021 in Denmark, Sweden, Japan, the Netherlands, Germany, the United Kingdom, Europe, Switzerland and the USA with recommendations for different types of tinnitus treatments, counseling and cognitive behavioral therapy were the only treatments unanimously recommended in all guidelines, whereas other modalities (e.g., tinnitus retraining therapy, sound therapy, hearing aids, cochlear implantation) were inconsistently recommended because of the low quality and quantity of the evidence reported in the individual studies [14].

Dietary supplements based on natural products may complement the treatment of patients with tinnitus and improve their quality of life (QoL). Antioxidants and cochlear vasodilators may support auditory well-being by enhancing circulation and protecting against oxidative stress. *Ginkgo biloba* is the most commonly used herbal supplement for tinnitus. In a systematic review based on five randomized controlled trials (RCTs), it was concluded that a standardized *Ginkgo biloba* extract is an evidence-based treatment option in tinnitus [15]. Minerals with antioxidant properties, such as zinc and selenium, have been shown to exert a clinically favorable effect on the subjective discomfort and tinnitus intensity in patients with low levels of these compounds [16,17]. Also, neurotropic vitamins B1, B6 and B12 are important for the maintenance of normal neurological functions in the peripheral and central nervous system, especially as cofactors in cellular energetic processes, antioxidative and neuroprotective effects, and both myelin and neurotransmitter synthesis [18,19]. Reduced intake of vitamins B12, B6 and B3 may be associated with tinnitus-related annoyance and different studies have shown improvement in symptoms after B-complex vitamins supplementation [20,21]. Melatonin has been successfully used to improve sleep quality in patients with tinnitus and sleep disturbances [22].

Additionally, low levels of *γ*-aminobutyric acid (GABA), an inhibitory neurotransmitter in the central auditory system maintaining excitation-inhibition balances, may contribute to tinnitus, and increasing GABA activity may help to create a calming effect, reducing stress and anxiety [23,24]. Finally, the health benefits of L-theanine, including reduction in stress and anxiety-like symptoms and improvements in mood, cognition, and sleep quality [25,26], would be of interest in the integral therapeutic approach of patients with tinnitus. L-theanine is able to pass the blood–brain barrier and exhibits pharmacological effects on brain wellness, increasing GABA level, enhancing dopamine release and modulating serotonin metabolism [27].

The present study was designed to assess the benefits of a natural dietary supplement containing L-theanine, GABA, *Ginkgo biloba*, zinc, selenium, melatonin, and B group vitamins (B3, B6 and B12) in patients diagnosed with unilateral or bilateral subjective tinnitus.

## 2. Materials and Methods

### 2.1. Design and Participants

A single-center observational open-label and prospective study was conducted at the Otorhinolaryngology and Head and Neck clinics of the AMiQ Medical and Surgical Group in Barcelona, Spain, between March and December 2024. All consecutive patients who met the eligibility criteria and provided written consent were included in the study. Inclusion criteria were age between 18 and 70 years old and diagnosis of persistent (≥6 months) unilateral or bilateral tinnitus. Exclusion criteria included the presence of significant audio-otologic diseases (Ménière’s disease, otosclerosis, tympanic perforation, or middle ear infection), acoustic neurinoma, otologic lesions/surgery of the inner ear, and pulsatile tinnitus; diagnosis of a psychiatric disorder such as depression or psychosis; severe renal failure; scheduled for elective surgery during the study; current treatment with antidepressant or anxiolytic drugs, and anticoagulants; pharmacological treatment for tinnitus within the previous 3 months; history of alcohol or drug abuse within the previous 6 months; consumption of tea (more than 4 cups daily); hypersensitivity to any component of the study product; pregnant or lactating women; participation in another clinical trial within the previous 6 months; and in-eligibility as judged by the investigators.

The study protocol was approved by the Clinical Research Ethics Committee of AMiQ-Institute of Ocular Microsurgery (IMO) (code 240115-250, approval date 26 June 2024), Barcelona, Spain. Written informed consent was obtained from all participants.

### 2.2. Intervention and Study Procedures

The investigational product was a dietary supplement containing L-theanine, GABA, *Ginkgo biloba*, melatonin, zinc, selenium, and vitamins B3, B6 and B12 (Otocalm^®^, Reig Jofré, Sant Joan Despí, Barcelona, Spain) authorized by the Spanish health authorities for improving auditory well-being. The composition of the product is shown in Table 1. The product contains two capsules that combine compounds whose mechanisms of action are optimized when taken at different times of the day to align with natural biological rhythms and maximize therapeutic benefit. Additionally, splitting the dose into two time points improves tolerability and absorption, minimizing potential gastrointestinal discomfort and avoiding competition between ingredients that may have different absorption pathways. It also ensures more stable physiological coverage throughout 24 h, supporting both daytime cognitive auditory processing and nighttime recovery.

Participants were instructed to take one capsule (lilac color) at the time of breakfast with a glass of water (200 mL) and one capsule (blue color) at night with dinner with a glass of water (200 mL). The duration of dietary supplementation was 90 days. It was strongly recommended not to make changes to the dietary habits and avoid taking more than four cups of tea daily, more than one cup of coffee daily and related products throughout the study.

Participants were visited at baseline (day 0, visit 1) and at the end of the study (day 90, visit 2). At the baseline visit, written informed consent was obtained, and fulfillment of the inclusion criteria was checked. Clinical assessments included a detailed medical history, physical examination, including otoendoscopy, tonal audiometry, and acufenometry (Madsen® Astera, Natus Medical Denmark ApS). Patients completed the Tinnitus Handicap Inventory (THI) as a measure of QoL, the Goldberg anxiety and depression scale (GADS), and evaluated the intensity of tinnitus using a visual analogue scale (VAS). Following the otorhinolaryngological evaluation, patients underwent a neurological assessment that included nutritional evaluation, laboratory tests, and an EEG under sleep deprivation. Participants were questioned about the sleep time, self-perceived level of stress, and consumption of caffeine, tobacco, alcohol, and performance of physical exercise on the day before EEG testing.

At the baseline visit, the study product for the entire study period was provided. At the end of the study (visit 2), the following procedures were performed: physical examination, THI, tonal audiometry, acufenometry, Goldberg anxiety and depression scale (GADS), VAS, and EEG. Participants were also interviewed about the tolerability of the study product and adverse events (AEs). The returned capsules at visit 2 (end of the study) were checked and counted for adherence with the study product.

### 2.3. Study Variables

Variables recorded included demographic data; body mass index (BMI); comorbidities; perceived tinnitus handicap severity using the THI [28], which is a 25-item self-report questionnaire with final score ranging from 0 to 100 and five grades of handicap (grade 1, scores 0–16: slight or no handicap; grade 2, scores 18–36: mild handicap; grade 3, scores 38–59: moderate handicap; grade 4, scores 58–76, severe handicap; grade 5, scores 78–100: catastrophic handicap); the GADS [29], which is a 18-item self-report questionnaire with two subscales of anxiety (9 items) and depression (9 items) and “yes” or “no” response options (higher point values indicate a more severe problem with 9 as the highest possible value for each subscale); 0–10 mm VAS score from 0: no tinnitus to 10: maximum tinnitus, with 0–3 score considered as tinnitus of mild intensity and minimal impact on activities of daily living (ADLs), 4–6 score as moderate intensity with moderate impact on ADLs, and 7–10 score as high intensity with severe impact on ADLs; tone verbal audiometry in which auditory capacity was classified as normal hearing, sensorineural hearing loss (left, right or bilateral), conductive hearing loss (left, right or bilateral), and mixed hearing loss; and acufenometry to assess the intensity (dB SL) and frequency (Hz) of tinnitus. Nutritional assessment included a series of questions to determine whether the participant followed a balanced diet without proinflammatory habits. Laboratory tests included a standard blood count, liver and renal function profiles, and serum levels of minerals and vitamins.

A 30-min conventional sleep-deprived EEG with 21 channels and the 10–20 electrode placement was used to record brain activity. N200 latency, P300 latency, and N200-P300 amplitude at the midline electrodes (Fz, Cz and Pz) were registered. Qualitative EEG data included rhythm (alpha: 8 to 12 Hz, beta: 13–30 Hz, theta: 4–7 Hz, and delta: 0–5 Hz waves) and amplitude (alpha: <50 μV, beta: 10–20 μV, theta: 10–30 μV, and delta: 50–100 μV). Quantitative EEG data included gamma rhythm (>30.80 Hz); temporal localization; posterior alpha rhythm (frequency 8–13 Hz, amplitude 15–20 μV); alpha, theta, beta, gamma, and delta power (μV); stimulus-selective response modulation (SRM); and global EEG coherence (1–30 Hz).

### 2.4. Study Endpoints

The primary endpoint was the improvement in emotional quality of life (QoL) using the GADS. Secondary endpoints were the reduction in the intensity and frequency of tinnitus measured by THI, VAS, and acufenometry, improvement in the auditory capacity, changes in EEG findings, and AEs.

### 2.5. Statistical Analysis

According to the exploratory nature of the study, a sample size of 30 participants was considered adequate. Categorical variables are expressed as frequencies and percentages, and continuous variables as mean and standard deviation (SD) with 95% confidence interval (CI). The Wilcoxon signed-rank test was used for the comparison of paired variables before and after 90 days of supplementation with the study product. A *p*-value < 0.05 was considered significant. The Statistical Analysis System (SAS Institute, Cary, NC, USA) version 9.4 was used for data analysis.

## 3. Results

### 3.1. Participants and Baseline Characteristics

A total of 30 eligible patients were included in the study. Six patients were visited at baseline but were lost to follow-up and 1 patient visited at baseline did not complete verbal tone audiometry. Therefore, at the end of the study, the otorhinolaryngological evaluation was completed by all 30 patients, the THI, GADS, and VAS score intensity for tinnitus by 24 patients, and verbal tone audiometry by 23 patients. Acufenometry and neurological evaluation were completed by 12 patients.

Clinical findings at the baseline visit are shown in Table 2. There were 15 men and 15 women with a mean (SD) age of 50.7 (11.3) years, and a mean BMI of 24.2 (3.7) kg/m^2^. Eighteen patients (60%) self-reported hearing loss, mostly of a mild degree. Clinical findings at the baseline visit are shown in Table 2. Tinnitus-related handicap according to the THI was grade 2 in 53.3% of patients, grade 3 in 23.3%, and grade 4 in 16.7%. One patient showed a catastrophic handicap. The mean score of the anxiety subscale of GADS was 4.8 (2.8), and 63.3% of patients scored ≥4, whereas the mean score of the depression subscale was 2.2 (1.9) and 56.7% of patients scored ≥2. The mean VAS score for tinnitus intensity was 6.5 (2.0). In the verbal tone audiometry, normal hearing was registered in 11 patients (36.7%), sensorineural hearing loss in 9 (30%), conductive hearing loss in 9 (30), and mixed hearing loss in 3 (10). The mean intensity of tinnitus was 42.9 dB SL, and the mean frequency was 2417.4 Hz.

In relation to the nutritional status, most patients (80%) reported following a healthy diet (i.e., not vegetarian, hypocaloric, vegan, etc.), and none exhibited proinflammatory dietary habits (i.e., refined carbohydrates, saturated fats, processed meats, etc.). On the day prior to EEG examination, patients reported a mean sleep duration of 6.5 (1.3) h. Stress levels were rated as moderate by 26.7% and severe by 20% of patients. Additionally, 66.7% consumed caffeine, 6.7% smoked, 13.3% consumed alcohol, and 6.7% had performed physical exercise. As shown in Table 3, EEG parameters were within normal ranges.

### 3.2. Data at the End of the Study

After 90 days of consumption of the dietary supplement (Table 4), the mean (SD) THI score was 30.9 (21.4), with grades 1 and 2 in 56.7% of patients. The mean score of the anxiety subscale of GADS was 3.0 (2.6) and 33.3% of patients scored ≥4, whereas in the depression subscale, the mean score was 1.7 (2.0) and 33.3% scored ≥2. The mean VAS score for tinnitus intensity was 6.1 (2.4). In the verbal tone audiometry, normal hearing was recorded in 29.2% of patients, sensorineural hearing loss in 37.5%, conductive hearing loss in 16.7%, and mixed hearing loss in 12.5%. The mean intensity of tinnitus was 42.5 dB, and the mean frequency was 1603.3 Hz. The results of EEG testing are shown in Table 5.

**Figure 1:**
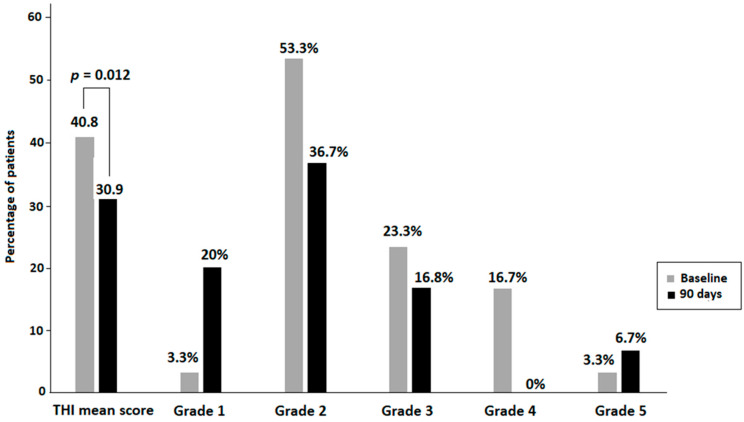
Improvement in the quality of life (QoL) according to the decrease in tinnitus-related handicap assessed with the Tinnitus Handicap Inventory (THI) at the end of the study (90 days) as compared with baseline.

**Figure 2:**
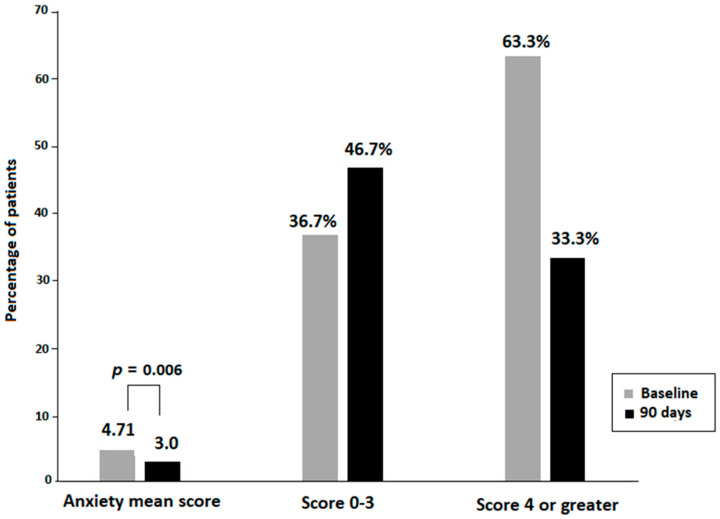
Improvement in the quality of life (QoL) related to decreases in the subscale of anxiety of the Goldberg anxiety and depression scale (GADS) (missed scores at 90 days for 6 patients).

The comparison of variables before and after consumption of the dietary supplement for 90 days showed statistically significant improvements in the mean THI score, with a difference of −9.17 (16.4) (*p* < 0.012). The percentages of patients with THI grades 1 also increased from 3.3% at baseline to 20% at the end of the study, with decreases in patients with grades 2 and 3 (Figure 1). In relation to QoL assessed by GADS, significant decreases in the subscale of anxiety at the end of the study were observed, with a mean difference of −1.71 (2.8) (*p* = 0.006). The percentage of patients with scores 0–3 also increased, whereas those with scores ≥ 4 decreased (Figure 2). Also, there were changes in some cognitive evoked potentials parameters including N200 latency at frontal midline (difference 63.9 [78.4] ms, *p* = 0.004) and parietal midline (difference 68.5 [84.5] ms, *p* = 0.013), P300 latency at frontal midline (difference 85.7 [87.4] ms, *p* = 0.001), central midline (difference 88.1 [116.9] ms, *p* = 0.018) and parietal midline (difference 110.4 [80.6], *p* = 0.002), and gamma rhythm (difference −10.6 [14.3] Hz, *p* = 0.007).

### 3.3. Tolerability and Safety

Treatment with the study product was well-tolerated. Only 8 patients reported minor AEs of mild intensity, such as occasional headache, abdominal complaints, and dizziness. Vital signs (pulse, blood pressure, respiratory rate) remained within normal limits, and results of laboratory tests were unrevealing. All patients were fully adherent to the study product.

## 4. Discussion

Results of the present study show that 90-day dietary supplementation with a natural-based product containing L-theanine, *Ginkgo biloba*, melatonin, GABA, zinc, selenium, and B group vitamins improved QoL in patients with tinnitus. Main findings of the study were statistically significant decreases in the mean scores of the THI questionnaire at the end of the study as compared with baseline, as well as significant reductions in the subscale of anxiety of the GADS instrument. In addition, improvement in QoL was also shown by increases in the percentages of patients with THI grade 1, indicating slight or no handicap related to tinnitus (from 3.3% at baseline to 20% at 90 days). Regarding anxiety evaluated as a 9-point score in the GADS self-reported questionnaire, the mean score of 4.7 at baseline decreased to 3.0 at the end of the study, with a mean percentage decrease of −30.8%. A remarkable finding was the decrease in the percentage of patients scoring ≥ 4 in the anxiety subscale from 63.3% at baseline to 33.3% at the end of the study. Reductions in the depression subscale were also observed, with a mean decrease of −33.8%, although differences as compared with baseline did not reach statistical significance.

Anxiety has a detrimental impact on QoL [30] and frequently develops as a consequence of tinnitus symptoms, rather than arising independently. The persistent perception of intrusive sound can trigger emotional distress, leading to anxiety that further affects QoL and activities of daily living. A large U.S. survey including 21.4 million adults with tinnitus showed that 26.1% reported problems with anxiety in the preceding 12 months, and individuals who described their tinnitus as a “big” or “very big” problem were significantly more likely to report anxiety and reduced sleep duration [31]. These findings indicate that tinnitus severity contributes to increase anxiety levels. Moreover, in a study that evaluated the link between tinnitus-related measures and general QoL questionnaires in 85 patients with tinnitus as their primary complaint, it was observed that while generic tools such as the Short-Form (SF-8) and the WHOQOL-BREF captured general physical, psychological, environmental, and social domains, they did not fully reflect the emotional burden induced by tinnitus [32]. In contrast, tinnitus-focused instruments such as the Tinnitus Functional Index (TFI) and THI are preferred in clinical practice because they more accurately assess the emotional distress—including anxiety—that arises in response to tinnitus [32].

The components of the natural-based dietary supplement, particularly L-theanine, *Ginkgo biloba*, GABA, and melatonin, have been shown to promote hearing health with improvement of tinnitus due to their anti-anxiety, relaxing, antioxidant, cochlear vasodilation, and other properties involved in the underlying neurophysiological mechanisms of tinnitus [15,16,17,18,19,20,21,22]. L-theanine, a unique free amino acid and one of the most important substances in green tea, is a bioactive compound with plenty of health benefits, including antioxidant, anti-inflammatory, neuroprotective, metabolic and immune regulatory effects [33]. These properties may be particularly valuable for individuals with tinnitus, as L-theanine can help to reduce anxiety and stress, which are factors known to exacerbate tinnitus symptoms. A systematic review of nine peer-reviewed journal articles showed that supplementation with green tea amino acid L-theanine may assist in the reduction of stress and anxiety in people exposed to stressful conditions [34]. However, as far as we are aware, no previous experience has reported the use of a dietary supplement containing L-theanine in the control of tinnitus. Other components of the dietary supplement, such as *Ginkgo biloba*, have shown beneficial effects in reducing tinnitus symptoms [35]. Interestingly, a recent study showed that *Ginkgo biloba* combined with an antioxidant preparation (β-carotene, vitamin C, vitamin E, and selenium) resulted in marked improvement in THI, VAS and QoL (assessed with the SF-36 health survey) as compared with *Ginkgo biloba* singly or placebo, suggesting a synergistic effect of *Ginkgo biloba* and antioxidants for treatment of tinnitus [36]. On the other hand, vitamins and minerals are associated with lower tinnitus risk [37], but the impact of the combination of selenium, zinc, B3, B6 and B12 vitamins on the QoL in patients with tinnitus has not been previously evaluated. In a study of 32 patients with tinnitus and concomitant headache, 90-day dietary supplementation with a formulation based on *Ginkgo biloba*, vitamin B12, magnesium, zinc, and melatonin was effective in reducing the symptoms of tinnitus and headache in patients suffering from both conditions [38], although in this study the composition of the food supplement was not reported and the potential effects of the product on QoL were not analyzed.

The dietary supplement used in the present study also contained melatonin and GABA. Melatonin alleviates subjective symptoms of tinnitus and has been extensively used to improve sleep quality [22,39], whereas enhancing GABA function targeting GABA-mediated neurotransmission in the auditory cortex is another mechanism to dampen symptoms of tinnitus [23]. In animal models of tinnitus, restoring central GABA down-regulation through increased GABA availability may eliminate evidence of tinnitus [40,41].

An interesting characteristic of the study product was the administration of its components in the form of two capsules at different times, one in the morning and the other at night. The different components of each capsule contribute to the most advantage of the anxiety and stress reducing effects of *Gingko biloba* and L-theanine at daytime, as well as the relaxing and sleep promoting effects of melatonin and GABA at nighttime. Supplementation with two capsules with different compositions based on the pharmacological characteristics, physiological effects, and chronobiological profiles of the active ingredients ensures synchronization with natural biorhythms and contributes to maximizing the therapeutic benefit.

In the subset of patients undergoing neurophysiological evaluation, a decrease in gamma rhythms and changes in cognitive evoked potentials with prolongation in both N200 and P300 latencies, and a decrement in N200-P300 amplitude were recorded, although interpretation of these findings is difficult due to the small sample of patients undergoing EEG examination. Other studies have shown differences in P300 latency and amplitude values in cases of sensorineural hearing loss with tinnitus when compared to normal hearing subjects, supporting the role of cognition and involvement of the central auditory pathway in tinnitus generation [11]. Also, P300 measurement may be a useful measurement in the evaluation of tinnitus therapies [12].

The present findings should be interpreted considering the limitations of the study, including the small sample size and the lack of a control group of patients treated with placebo. Also, only half of the study sample underwent acufenometry and neurological evaluation. In addition, it may be possible that an extended duration of the dietary supplementation would increase the benefits on QoL already observed after 3 months of supplementation. The study was an exploratory investigation to generate preliminary evidence of the benefits of the multicomponent product, based on different active ingredients administered twice a day to enhance the pharmacological actions of each individual component in accordance to natural biorhythms. The beneficial effects obtained in tinnitus-related handicap and anxiety variables give rise to carry out further randomized placebo-controlled studies with larger study populations.

## 5. Conclusions

The administration of a natural-based dietary supplement composed of L-theanine, *Ginkgo biloba*, GABA, melatonin, zinc, selenium, and vitamins B3, B6 and B12 for 90 days in patients with tinnitus was associated with beneficial effects significantly increasing QoL by reducing tinnitus-associated handicap and anxiety. Treatment was safe and well-tolerated. Given the annoying characteristics of persistent tinnitus, its negative impact on QoL, and the fact that tinnitus treatment is often disappointing, results of the present exploratory study are promising, but further evaluation of this dietary intervention in the framework of a placebo-controlled randomized study with a larger study population is needed.

## Figures and Tables

**Table 1:** Composition of the study product.

Ingredient	Daily Dose	Daily Recommended Dose
Morning capsules		
Gingko biloba (plant leaves extract: GINKGO LEAF PE 4:1 REF. NO-EC856117)	54 mg	120–240 mg
Zinc	5 mg	8–11 mg
Selenium	35 μg	55–70 μg
L-theanine	60 mg	50–450 mg
Vitamin B3	16 mg	14–16 mg
Vitamin B12	25 μg	50–500 μg
Night capsules		
Melatonin	1.9 mg	0.5–5 mg
GABA	250 mg	50–3000 mg
Vitamin B6	0.7 mg	1.3–1.7 mg
Vitamin B12	25 μg	50–500 μg
Selenium	35 μg	55–70 μg

GABA: *γ*-aminobutyric acid.

**Table 2:** Baseline clinical findings in 30 patients with tinnitus.

Variables	Number (%)
Gender	
Men	15 (50)
Women	15 (50)
Age, years, mean (SD)	50.7 (11.3)
Body mass index (BMI), kg/m^2^, mean (SD)	24.2 (3.7)
Self-reported hearing loss	18 (60)
Mild	11 (36.7)
Moderate	5 (16.7)
Severe	2 (6.7)
Comorbidities	
Allergy	13 (43.3)
Hypertension	3 (10)
Chronic gastritis	1 (3.3)
Irritable bowel syndrome	1 (3.3)
Hip arthrosis	2 (6.7)
Cutaneous eczema	1 (3.3)
Anemia	1 (3.3)
Fatty liver	1 (3.3)
Peripheral vascular disease	1 (3.3)
Otosclerosis right ear	1 (3.3)
Tinnitus Handicap Inventory (THI), mean (SD)	40.1 (18.5)
Grade 1	1 (3.3)
Grade 2	16 (53.3)
Grade 3	7 (23.3)
Grade 4	5 (16.7)
Grade 5	1 (3.3)
Goldberg anxiety and depression scale (GADS)	
Anxiety subscale, mean (SD)	4.8 (2.8)
Total score 0–3	11 (36.7)
Total score > 4	19 (63.3)
Depression subscale, mean (SD)	2.2 (1.9)
Total score 0–1	13 (43.3)
Total score > 2	17 (56.7)
VAS score intensity of tinnitus, mean (SD)	6.6 (2.0)
Verbal tonal audiometry	
Normal hearing	11 (36.7)
Sensorineural hearing loss	
Right	2 (6.7)
Left	3 (10)
Bilateral	4 (13.3)
Conductive hearing loss	
Right	0
Left	7 (23.3)
Bilateral	2 (6.7)
Mixed hearing loss	3 (10)
Tinnitus intensity, dB SL, mean (SD)	42.9 (14.1)
Tinnitus frequency, Hz, mean (SD)	2417.4 (2485.1)

Acufenometry was performed in 15 patients. Data expressed as frequencies and percentages in parenthesis unless otherwise stated.

**Table 3:** Baseline electroencephalographic (EEG) findings in 15 patients with tinnitus.

Data	Mean (SD)	95% Confidence Interval
Cognitive evoked potential		
N200 latency, ms		
Fz	146.0 (52.6)	116.9–175.1
Cz	149.6 (56.3)	117.1–182.1
Pz	168.9 (70.4)	128.3–209.6
P300 latency, ms		
Fz	198.5 (55.2)	168.0–229.1
Cz	199.5 (61.3)	164.1–234.9
Pz	208.1 (77.8)	163.2–253.0
N200-P300 amplitude, μV		
Fz	5.5 (9.8)	0.05–10.9
Cz	2.8 (2.1)	1.6–4.0
Pz	2.6 (2.2)	1.4–3.9
Qualitative EEG		
Rhythm		
Alpha wave, Hz	9.8 (0.4)	9.6–10.0
Beta wave, Hz	14.5 (1.1)	13.9–15.1
Theta wave, Hz	4.6 (0.6)	4.3–4.9
Delta wave, Hz	3.1 (3.6)	1.1–5.1
Amplitude		
Alpha wave, Hz	18.4 (15.6)	9.8–27.1
Beta wave, Hz	6.9 (2.8)	5.3–8.4
Theta wave, Hz	7.4 (2.9)	5.8–9.0
Delta wave, Hz	9.1 (5.1)	6.3–11.9
Quantitative EEG *^,†^		
Gamma rhythm, Hz	33.2 (3.4)	31.3–35.2
Posterior alpha rhythm		
Frequency, Hz	9.8 (0.4)	9.5–9.9
Amplitude, μV	17.8 (15.8)	9.0–26.5
Alpha power, μV	1.0 (0.2)	0.1–0.2
Theta power, μV	0.05 (0.03)	0.03 (0.07)
Beta power, μV	0.08 (0.06)	0.05–0.11
Gamma power, μV	0.07 (0.10)	0.01–0.12)
Delta power, μV	0.07 (0.07)	0.03 (0.11)
Coherence, Hz	18.7 (15.8)	−6.3–43.8

* Temporal localization in 100% of patients; ^†^ stimulus-selective response modulation (SRM): present in 33.3% of cases, absent in 66.8%; ms: milliseconds; Fz: frontal midline; Cz: central midline; Pz: parietal midline; μV: microvolts; Hz: hertz.

**Table 4:** Results of otorhinolaryngological examination in 24 patients at the end of the study.

Variables	Number (%)
Tinnitus Handicap Inventory (THI), mean (SD)	30.9 (21.4)
Grade 1	6 (20)
Grade 2	11 (36.7)
Grade 3	5 (16.7)
Grade 5	2 (6.7)
Missing	6 (20)
Goldberg anxiety and depression scale (GADS)	
Anxiety subscale, mean (SD)	3.0 (2.6)
Total score 0–3	14 (46.8)
Total score > 4	10 (33.3)
Depression subscale, mean (SD)	1.7 (2.0)
Total score 0–1	14 (46.7)
Total score > 2	10 (33.3)
VAS score intensity of tinnitus, mean (SD)	6.1 (2.4)
Verbal tonal audiometry (*n* = 23)	
Normal hearing	7 (29.2)
Sensorineural hearing loss	
Right	2 (8.3)
Left	3 (12.5)
Bilateral	4 (16.7)
Conductive hearing loss	
Right	0
Left	2 (8.3)
Bilateral	2 (8.3)
Mixed hearing loss	3 (12.5)
Acufenometry (*n* = 12)	
Tinnitus intensity, dB, mean (SD)	42.5 (17.5)
Tinnitus frequency, Hz, mean (SD)	1603.4 (2374.9)

Data expressed as frequencies and percentages in parenthesis unless otherwise stated.

**Table 5:** Electroencephalographic (EEG) findings in 12 patients at the end of the study.

Data	Mean (SD)	95% Confidence Interval
Cognitive evoked potential		
N200 latency, ms		
Fz	207.3 (70.3)	162.7–252.0
Cz	209.9 (71.2)	164.7–255.2
Pz	227.1 (70.2)	182.5–271.7
P300 latency, ms		
Fz	284.7 (74.5)	237.4–332.1
Cz	286.2 (80.6)	235.0–337.4
Pz	308.8 (78.5)	259.0–358.7
N200-P300 amplitude, μV		
Fz	4.4 (2.2)	3.0–5.8
Cz	2.2 (1.4)	1.3–3.1
Pz	2.0 (1.5)	1.1–3.0
Qualitative EEG		
Rhythm		
Alpha wave, Hz	9.6 (0.6)	9.2–10.0
Beta wave, Hz	14.5 (0.6)	14.1–14.9
Theta wave, Hz	4.8 (0.6)	4.4–5.2
Delta wave, Hz	1.9 (0.3)	1.6–2.1
Amplitude		
Alpha wave, Hz	15.8 (10.4)	9.3–22.4
Beta wave, Hz	6.7 (3.6)	4.4–8.9
Theta wave, Hz	6.6 (2.7)	4.8–8.3
Delta wave, Hz	7.9 (4.4)	5.1–10.6
Quantitative EEG *^,†^		
Gamma rhythm, Hz	23.4 (15.1)	13.2–33.5
Posterior alpha rhythm		
Frequency, Hz	9.5 (0.5)	9.2–9.8
Amplitude, μV	12.7 (10.8)	5.8–19.5
Alpha power, μV	0.14 (0.11)	0.07–0.21
Theta power, μV	0.07 (0.04)	0.04 (0.09)
Beta power, μV	0.11 (0.11)	0.05–0.18
Gamma power, μV	0.03 (0.02)	0.02–0.04)
Delta power, μV	0.07 (0.08)	0.01 (0.12)
Coherence, Hz	14.0 (0)	

* Temporal localization in 8 patients (66.7%) (missing 4 patients); ^†^ stimulus-selective response modulation (SRM): present in 16.7% of cases, absent in 83.3%; ms: milliseconds; Fz: frontal midline; Cz: central midline; Pz: parietal midline; μV: microvolts; Hz: hertz.

## Data Availability

The data that support the findings of this study are not publicly available due to privacy reasons but are available from the corresponding author upon request.

## Abbreviations

The following abbreviations are used in this manuscript:

ADLs	Activities of daily living
AEs	Adverse events
BMI	Body mass index
CI	Confidence interval
Cz	Central midline
EEG	Electroencephalography
Fz	Frontal midline
GABA	Gamma-aminobutyric acid
GADS	Goldberg anxiety and depression scale
Hz	Hertz
ms	milliseconds
Pz	Parietal midline
QoL	Quality of life
RCT	Randomized controlled trial
SD	Standard deviation
SRM	Stimulus-selective response modulation
THI	Tinnitus Handicap Inventory
TFI	Tinnitus Functional Index
VAS	Visual analogue scale
μV	Microvolts
WHOQOL-BREF	World Health Organization Quality of Life
